# ANXIETY AND DEPRESSION IN TUNISIAN PATIENTS WITH PARKINSON’S DISEASE

**DOI:** 10.1192/j.eurpsy.2023.2041

**Published:** 2023-07-19

**Authors:** S. Kolsi, E. Fakhfakh, N. Messedi, I. Chaari, F. Charfeddine, L. Aribi, N. Farhat, C. Mhiri, J. Aloulou

**Affiliations:** 1 Psychiatry Department B, Hedi Chaker Hospital Sfax, Tunisia; 2 Neurology Department, Hbib Bourguiba Hospital, Sfax, Tunisia

## Abstract

**Introduction:**

Anxiodepressive disorders in Parkinson’s disease (PD) are quite frequent and considered as non-motor signs of the disease. Few studies have studied the link between these entities.

**Objectives:**

The objective of this study is to determinate the prevalence of anxiety and depression in patients with PD, and factors associated with them

**Methods:**

A descriptive and analytical cross-sectional study collected from patients followed at the neurology consultation of Habib Bourguiba’s university Hospital in Sfax, Tunisia. We used:

- a sociodemographic, clinical and therapeutic data sheet
- Hospital Anxiety and Depression Scale(HADS): to study anxiety and depression. A score ≥10 means a certain anxiety or depression

**Results:**

We have involved 47 patients. The average age was 61.47 years with a sex ratio (M/W)=1,47. Amantadine was received by 12.77% of patients, anticholinergics by 14.9% of patients and 87.24% of patients were treated with Levodopa.

HADS: the average scale of Depression was8.62 ± 4.54 and the median scaleanxiety was 7 [1-18]. Depression and anxiety were presentin 38.29% and 27.68% of cases, respectively. Depression was significantly correlated with Amantadine intake (P=0.036) and Levodopa dose (P=0.016). Anxiety was significantly associated with anticholinergic intake(P=0.011).Both depression and anxiety were notstatistically correlated to the presence ofmotor complications of dopa therapy (P= 0.44 and 0.7 respectively)

**Conclusions:**

The therapeutic management of patients with PD influences the occurrence of anxiety and depression, which proves the importance of early detection of these disordersto ensure better support

**Disclosure of Interest:**

None Declared

